# Dr. Bela H. Colgrove

**Published:** 1874-03

**Authors:** 


### Dr. Bela H. Colegrove.

A special meeting of the Erie County Medical Society was held Saturday evening March 21st, to take into consideration the death of Dr. B. H. COLEGROVE, of Sardinia.

The President of the Society, Dr. LOTHROP upon taking the chair, stated in a few words the object of the meeting. Dr. JAMES P. WHITE made a few remarks concerning the deceased, and gave a brief account of his acquaintance with him.

Dr. LYNDE read the following brief history of Dr. COLEGROVE'S professional career.

"No man in our profession can, through nobility of birth, fortune or patronage, attain to eminence. Neither is there any chance in its ranks for accidental fame. Its laws in this respect are inexorable. Only those can hope for distinction who possess integrity, natural abilities, discriminating powers, and untiring industry. A small man may creep into high places in the political world, and his death in consequence may shock a continent. A great man may fall from the ranks of our profession without eliciting anything more than the common obituary notice, which from the most of its readers but calls out the interrogation, Who was he? Who then was he? He was Bela H. Colegrove, of the little village of Sardinia, where for over half a century he lived the counsellor alike of medical men and the people; and I venture the assertion that no member of our profession ever met and conversed with Dr. Colegrove on medical topics but felt that he had profited thereby, and that no man outside of our profession ever formed his acquaintance but recognized his equal; and just here let me state that the deceased was associated in years gone by with men of note—judges, lawyers, congressmen and presidents

Dr. Colegrove, like all country practitioners, was obliged to cover the whole field of practice, including surgery, medicine and obstetrics, but unlike most men spread over so much territory, he was never regarded by his professional brethren as decidedly vulnerable at any point. So far was he from being feeble, especially in surgery, that during a professional life of over half a century, in which time he performed all or nearly all the recognized surgical operations of his day, (alone so far as responsibility was concerned) he never was mulcted for damages or took the part of defendant in the court-room. Mr. President—No one who has not the experience can know what it is to be a general practitioner in the country. I have no reference now to physical hardships, but I refer to the great responsibilities the practitioner is often driven to assume unaided by professional men, and often without a choice of surgical applicances or instruments. All this is reversed in city practice.

Dr. Colegrove was born in Coventry, R. I., on the 20th of April, 1797, and died in Sardinia March 19, 1874, making him at the time of his death nearly 77 years of age. His parents were poor, and he commenced life by teaching school at the age of 14. He follow teaching several years, saving his earnings which he afterwards expended in acquiring a knowledge of his profession He studied in the office of Dr. Hubbard, who was subsequently made professor of surgery in the New Haven Medical College.

He had as one of his fellow-students, while in the office of Dr. Hubbard, George McClellan, who in after years became professor of surgery in the Jefferson Medical College, Philadelphia. Dr. Colegrove attended his first course of lectures in the New Haven Medical College, his second in the Pennsylvania University. He had now spent less time in the study of his profession than was required by the curriculum of that old institution, and in company with three young men, similiarly situated, he started for his home in Rhode Island. On his way home he and his companions stopped in New York City, where he learned he could get an examination by the professors of the College of Physicians and Surgeons, and if they judged him competent a diploma would be given to him. All four of these young men were examined by the professors separately and but one rejected. Young Colegrove was among the fortunate, and received his diploma, which he had recorded in the Erie County Clerk's office, of this State, in 1821, making him at the time of his death a member of this society fifty-three years.

Dr. Colegrove began the practice of his profession in Sardinia, in this county, in 1820. He immediately entered into a good practice, which extended during his best days over half a dozen counties in this State, often being called into the northwestern counties of Pennsylvania. He entered into a copartnership with Dr. Trowbridge, of Buffalo, and moved to this city with a view of making it his home. He lived here but a few months and returned to his old home in Sardinia. Dr. Colegrove was not, however entirely contented in Sardinia. He at one time had his goods on the road to Springville, but for some cause changed his mind and directed his teamsters to return. Dr. Colegrove was strictly a professional man, and during his active life, such was his vigor, energy and industry, that, although in his saddle most of the time day and night, he kept well informed on all subjects that relate to his profession. He was proud and desirous of the high esteem of his fellow-practitioners.

He loved his profession, and was proud of it until the day of his death. These sentiments his profession have always reciprocated. But he was proud, too, of the admiration of the people, which led him to accept several elective offices. The duties of these offices compelled him to mar the otherwise continual, honorable, I might say, glorious service in his profession.

The chair then appointed a committee of three, consisting of Drs. WHITE, LYNDE and BRIGGS, to draft resolutions expressive of the feeling of the Society. They presented the following which were adopted:

*Whereas*, It has pleased Almighty God in His providence to remove from earth the soul of our aged friend and co laborer, BELA H. COLEGROVE, M. D.; therefore be it

*Resolved*, That by the death of Dr COLEGROVE the medical profession has lost one of its brightest ornaments and most zealous members; the State a pure, noble-minded and useful citizen, and the county in which he lived an able and disinterested advocate of its interests.

*Resolved*, That the Erie County Medical Society, upon the occasion of the death of its oldest and one of its ablest members, place on record its appreciation of a life devoted for over half a century to the most self-sacrificing labors and to the accomplishment of high professional ends.

*Resolved*, That this Society regards his long and honorable career with feelings of deep emotion, and hereby expresses its profound sense of gratitude to Almighty God for His gracious preservation of a life devoted to works of charity and love.

*Resolved*, That we tender our sympathy and condolence to the family of the deceased in their sad bereavement, and especially invoke upon them the Divine blessing.

*Resolved*, That a copy of these resolutions be furnished the Buffalo *Medical and Surgical Journal* and the daily press for publication, and that a copy be sent to his family.

After some remarks by various members of the Association the chair appointed a committee of five to attend the funeral on the part of the Society, after which the meeting was declared adjourned.

